# Characteristics of Wall Shear Stress and Pressure of Intracranial Atherosclerosis Analyzed by a Computational Fluid Dynamics Model: A Pilot Study

**DOI:** 10.3389/fneur.2019.01372

**Published:** 2020-01-17

**Authors:** Zimo Chen, Haiqiang Qin, Jia Liu, Bokai Wu, Zaiheng Cheng, Yong Jiang, Liping Liu, Lina Jing, Xinyi Leng, Jing Jing, Yilong Wang, Yongjun Wang

**Affiliations:** ^1^Department of Neurology, Beijing Tiantan Hospital, Capital Medical University, Beijing, China; ^2^China National Clinical Research Center for Neurological Diseases, Beijing, China; ^3^Center of Stroke, Beijing Institute for Brain Disorders, Beijing, China; ^4^Beijing Key Laboratory of Translational Medicine for Cerebrovascular Disease, Beijing, China; ^5^Shenzhen Institutes of Advanced Technology, Chinese Academy of Sciences, Shenzhen, China; ^6^Department of Radiology, Beijing Tiantan Hospital, Capital Medical University, Beijing, China; ^7^Department of Medicine and Therapeutics, The Chinese University of Hong Kong, Prince of Wales Hospital, Shatin, China

**Keywords:** intracranial atherosclerosis, cerebral hemodynamics, wall shear stress, pressure, mathematical modeling, magnetic resonance angiography

## Abstract

**Background:** Although wall shear stress (WSS) and pressure play important roles in plaque vulnerability, characteristics of the two indices in intracranial atherosclerosis (ICAS) have not been fully investigated yet. This study aimed to elucidate this issue by means of establishing a non-invasive computational fluid dynamics method with time-of-flight magnetic resonance angiography (TOF-MRA) of the whole cerebral artery.

**Materials and Methods:** Subjects with symptomatic ICAS in the middle cerebral artery domain were enrolled, excluding those with concomitant internal carotid artery stenosis. Based on patient-specific TOF-MRA images for three-dimensional (3D) meshes and arterial blood pressure with patient-specific carotid artery ultrasonography for inlet boundary conditions, patients' three-dimensional hemodynamics were modeled by a finite element method governed by Navier-Stokes equations.

**Results:** Among the 55 atherosclerotic lesions analyzed by this TOF-MRA based computational fluid dynamics model, the maximum WSS (WSS_max_) was most frequently detected at the apex points and the upper half of the upstream sections of the lesions, whereas the maximum pressure was most often located at the lower half of the upstream sections. As the percent stenosis increases, the relative value of WSS_max_ and pressure drop increased with significantly increasing steep beyond 50% stenosis. Moreover, WSS_max_ was found to linearly correlate with pressure drop in ICAS.

**Conclusions:** This study on ICAS revealed certain trends of longitudinal distribution of WSS and pressure and the influences of percent stenosis on cerebral hemodynamics, as well as the correlations between WSS and pressure drop. It represents a step forward in applying computational flow simulation techniques in studying ICAS and stroke, in a patient-specific manner.

## Introduction

Globally, stroke is a leading cause of mortality, disability, and the economic costs of treatment (1). Therein, intracranial atherosclerosis (ICAS) has been recognized as one of the most common causes of ischemic stroke and accounts for a majority of stroke recurrence, contributing to 30–50% of ischemic stroke and transient ischemic attack in Asian (2, 3). Despite optimal medical treatment, the remaining high risk of stroke in ICAS patients still lies in the limited understanding of underlying pathogenesis and that pathophysiological significance of ICAS cannot be completely reflected by anatomical severity, especially for mild and moderate stenosis (4).

One of the potential mechanisms is identified as the destabilizing effect by hemodynamic forces acting on plaques, generated by cerebral blood flow, the major indices of which are wall shear stress (WSS) and pressure (5). Thus, high mechanical load generated by hemodynamic forces often reveals a hemodynamic pattern prone to plaque rupture. In theory, high WSS may impose higher risk of plaque rupture, whereas low WSS is associated with the formation of plaques. In addition, pressure and the resultant translesional “pressure drop” also significantly affect the plaque vulnerability due to its considerable contribution to the total mechanical load as compared with WSS (5). It is therefore of great significance to clarify the characteristics of WSS and pressure of ICAS, in view of risk evaluation. Despite these speculations, data remain scarce by far on this issue.

Several studies have applied computational fluid dynamics (CFD) to simulate and analyze the hemodynamics of ICAS (6–8). However, these studies relied on images of computed tomography angiography, which causes concerns in radioactivity and contrast administration, and applied with the hypothetical inlet flow condition. In contrast, time-of-flight magnetic resonance angiography (TOF-MRA) is both radiation- and contrast-free (9, 10).

Thus, in this study, we aimed to investigate the characteristics of WSS and pressure through the non-invasive CFD model based on TOF-MRA and patient-specific inlet flow condition.

## Materials and Methods

### Patients

Patients were screened and selected from a registry study conducted in our center. Subjects aging 18–80 years with symptomatic ICAS in middle cerebral artery (MCA) domain within 7 days after the symptom onset were included, excluding those with concomitant internal carotid artery stenosis. All subjects must have finished the examinations of brain TOF-MRA and carotid artery ultrasonography, with velocity and diameter information available. Percent stenosis on TOF-MRA was calculated according to Warfarin-Aspirin Symptomatic Intracranial Disease trial (11). This cross-sectional study was approved by the ethics committee of Beijing Tiantan Hospital according to the principles expressed in the Declaration of Helsinki. All patients signed a written informed consent form.

### MRI

Three-dimensional (3D) TOF-MRA was performed with a 3.0T MR scanner (Trio-Tim; Siemens, Erlangen, Germany), equipped with a maximal slew rate of 200 mT·m^−1^·ms^−1^, a maximal gradient strength of 45 mT/m, a repetition time/echo time of 28/3.04 ms, a field of view of 20 × 18 cm^2^, matrix of 256 × 179, thickness of 0.7 mm, slices per slab of 40, and flip angle of 13°.

### CFD Modeling

A personal workstation was used to process source images of TOF-MRA, and the data were stored in standard Digital Imaging and Communications in Medicine format. The 3D geometric model was then reconstructed by a suite of software, Mimics (Materialize NV, Belgium), and then examined and manually revised by two neurologists, with the primary collaterals included. Further processing of the vascular surface and generation of the computational domain (volume mesh) was performed using the ANSYS ICEM CFD meshing software (ANSYS, Inc., USA) (6–8).

Considering the complexity of the cerebral artery geometry, we used the unstructured tetrahedral cell for domain discretization, and the mesh was finer nearby the area of stenosis where the flow field was of greater interest (6). The total number of elements was above one million, and the minimum volume of the elements was about 1.0 × 10^−8^ cm^3^ to capture the small-scale feature of the blood flow. The blood flow was assumed to be a viscous and incompressible Newtonian fluid. The blood parameters were defined by constant density ρ = 1.06 × 10^−3^ kg·m^−1^ and constant dynamic viscosity μ = 3.5 × 10^−3^ kg·m^−1^·s^−1^.

Based on the data of carotid artery ultrasonography, we calculated the blood flow rate as the inlet condition with the formula: blood flow rate = mean velocity × cross-sectional area, where mean velocity was obtained using the value: 1/3 peak-systolic velocity + 2/3 end-diastolic velocity. We then applied an instantaneous pulsatile inflow and incorporated a lumped parameter model to mimic the downstream effects from the distal vessels on the outlet boundaries. The detailed calculation of the outlet condition was performed by the same method reported previously (8). The governing equations of blood flow were described by 3D steady incompressible Navier-Stokes equations. This model was solved by a parallel computer with 240 CPU cores using the Newton-Krylov-Schwarz method.

**Figure 1:**
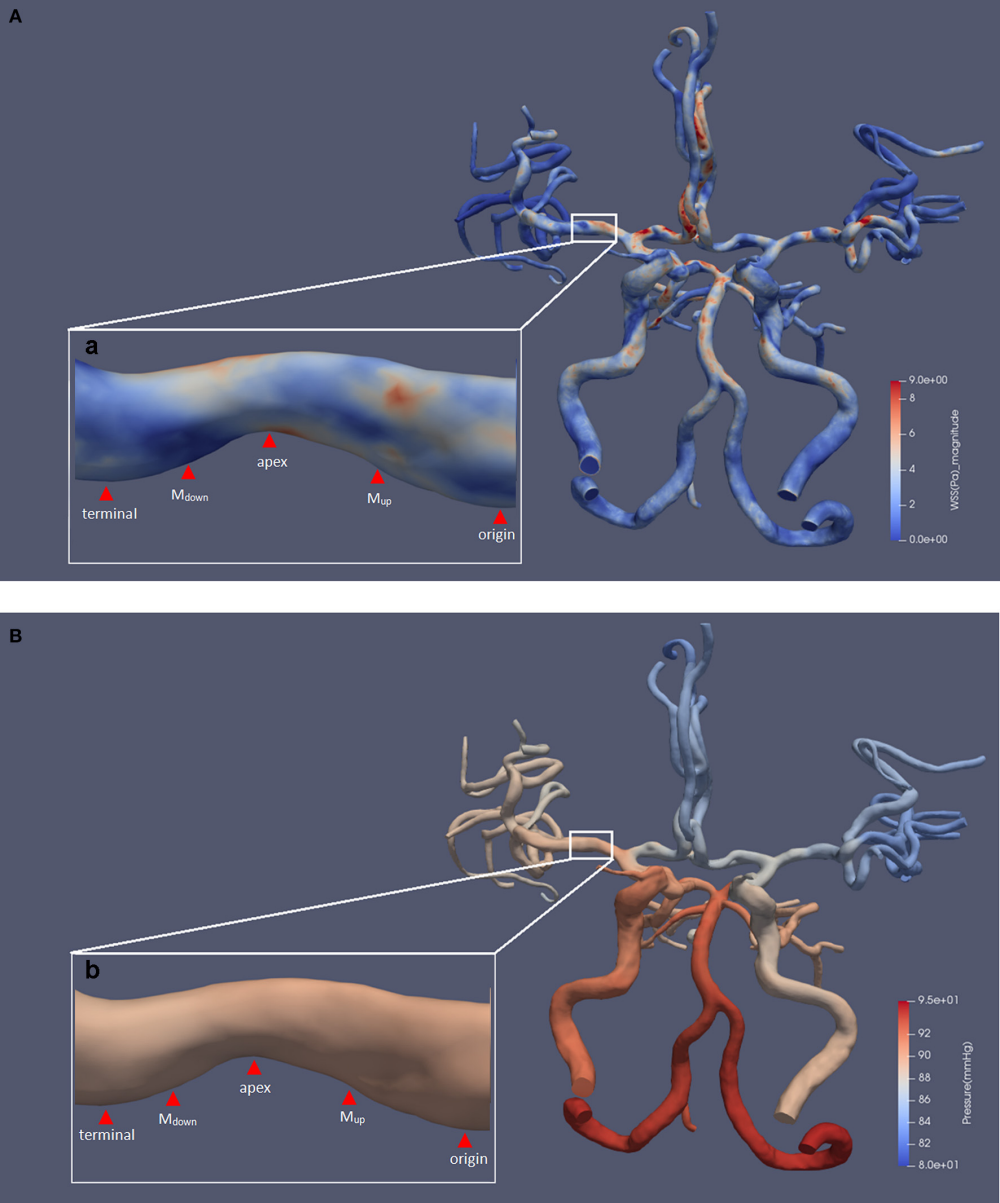
The results of a typical hemodynamic simulation. The five defined points of the prominent side are shown. Panels **(A)** and **(B)**, respectively, show the results of WSS and pressure contour maps. Subfigures (a) and (b) show an area of 34.3% luminal stenosis in the right MCA M1 segment. We chose the posterior wall of the MCA as the prominent side of the lesion for measurement. The WSS_max_ was located at the upper half of upstream section, and the magnitude was 7.47 pa. The WSS_min_ was located at the downstream section, and the magnitude was 0.33 pa. The pressure ratio_(termianl/origin)_ was 0.99. MCA, middle cerebral artery; WSS, wall shear stress; WSS_max_, the maximum value of WSS; WSS_min_, the minimum value of WSS; pressure_terminal_, the value of pressure at the terminal point; pressure_origin_, the value of pressure at the origin point.

An example of the hemodynamic simulation is shown in Figure 1. In this case, we included the bilateral anterior cerebral arteries and the posterior arteries into the CFD model where anterior communicating artery and right posterior communicating artery can be observed from the TOF-MRA scan.

### Measurements and Definition of the Specific Indices of WSS and Pressure

We used ParaView software (5.5.0 64-bit) (Kitware Inc., USA) for extraction and measurement of WSS and pressure from the simulated CFD models. For each lesion, the measurement was performed at the most severely narrowed longitudinal section of the diseased MCA, as shown in Figure 1, in accordance with previous studies on carotid and coronary arteries (12, 13). Then, the prominent sides of lesions were defined by a greater contribution to the total luminal stenosis compared with the other side. We defined five measuring points along the longitudinal axis of the prominent side, as shown in Figure 1. The first and the last measuring points were located at the entrance and exit of the stenosis and were defined as the origin point (origin) and the terminal point (terminal). The tip of the lesion was then defined as the apex. Two more measuring points were located at the middle point of the origin-to-apex and the apex-to-terminal sections, which were defined as “the middle point of upstream section” (M_up_) and “the middle point of downstream section” (M_down_), respectively. We further defined the maximum and minimum values of WSS and pressure on the prominent side as WSS_max_, WSS_min_, pressure_max_, and pressure_min_, respectively.

Due to the inlet flow conditions estimated on a patient-specific basis, the consequent hemodynamic parameters are determined by inlet flow conditions partly, which could cause difficulty in analyzing their relations with percent stenosis under the individualized inlet flow conditions. We therefore normalized them to achieve the relative indices such as WSS ratio_(max/origin)_ and pressure ratio_(terminal/origin)_, divided by the value of corresponding origin point, in order to attenuate the individual variation. In addition, we also used the indices of pressure drop_(origin-*to-terminal*)_ to gauge the absolute changes of pressure across the entire lesion, calculated as pressure_origin_ subtracted by pressure_terminal_.

All measurements, as shown in Figure 1, were performed by two experienced neurologists, and the values were then averaged. In order to minimize the possible hemodynamic interferes, tandem lesions with in-between distance <2 cm and lesions located at the arterial bifurcation or at the opening of a branch were excluded from the measurements (14, 15).

### Statistical Analysis

Categorical variables were expressed as absolute or relative frequencies. Age and percent stenosis were described using the means ± SD. Indices of WSS and pressure were described using medians (interquartile range). The kappa statistics was performed to calculate the intra/inter-observation agreement between the two neurologists for the identification of atherosclerotic lesions. To compare WSS, pressure, and pressure drop between different locations, we used the Friedman Test for repeated measurements and the Bonferroni correction for multiple comparisons. Comparisons of proportions were calculated using the chi-square test. Spearman's rank correlation was applied to evaluate the correlations between percent stenosis and specific indices, as well as the correlations between indices of hemodynamic forces, with adjustment of age and sex.

Two-sided *p*-values of 0.05 were considered statistically significant. All analyses were performed with SAS 9.4 (SAS Institute, Cary, NC, USA).

## Results

Overall, 55 lesions within the MCA domains selected from 22 patients (mean age, 65.5 years; 54.5% males) were included into the current analysis. For identification of atherosclerotic lesions, the intra/inter-observation agreements between the two neurologists were 0.877 and 0.815, respectively. The mean value of the percent stenosis of these lesions was 43.2% (±17.3%). Specifically, 26 (47.3%) lesions were located in M1 segments, and 24 (43.6%) lesions were percent stenosis ≥50%. Patients' demographic information is shown in Table S1.

### Description of the Magnitude of WSS and Pressure in Defined Points and Sections

WSS and pressure were longitudinally asymmetric across lesions in general, and the variability of the indices at each location was remarkable (Table S2). The Friedman Test showed that the differences of magnitude between indices of different locations were significant in general (*p* < 0.001). WSS_apex_ was the highest among the five points, followed by WSS_Mup_ (Table S3). In terms of pressure, it dropped significantly from M_up_ to M_down_, with non-significant change between origin and M_up_, as well as M_down_ and Terminal (Table S4). Accordingly, pressure drop from M_up_ to M_down_ [pressure drop_(Mup-*to-apex*)_ and pressure drop_(apex-*to-Mdown*)_] were the largest among the entire prominent side, with non-significant differences between each other (Table S5).

### Distribution of Specific Indices of WSS and Pressure

The WSS_max_ was most commonly found at the apex points (52.7%) and was also frequently located at the upper half of upstream sections (M_up_-to-apex) (40.0%), with no significant difference of this feature between the two subgroups of stenosis severity (<50, ≥50%). On the contrary, WSS_min_ was commonly observed at the downstream sections and the terminal points (38.2 and 36.4%, respectively), and it was also observed at the origin points (20.0%).

The pressure_max_ was almost all located at the origin points and the lower half of upstream sections (67.3 and 27.3%, respectively). Similar to WSS, the major location of pressure_min_ was within the downstream sections (70.9%) and terminal points (25.5%). These observations were consistent across the two subgroups of stenosis severity. The results of statistical analysis and detailed information on distribution are shown in Table 1.

**Table 1:** Distribution of specific indices at defined points and sections (*p*-values for the results of chi-square test indicate whether the most common location of the total distribution becomes significantly different after grouping).

**Specific indices of hemodynamic forces**	**Defined points and sections**	**Total**	**Grouped by stenosis severity**		***p-*value**
			**Percent stenosis ≥50%**	**Percent stenosis <50%**	
WSS_max_	Apex	29 (52.7%)	14 (58.3%)	15 (48.4%)	0.464
	M_up_-to-apex	22 (40.0%)	9 (37.5%)	13 (41.9%)	
	M_down_	3 (5.5%)	1 (4.2%)	2 (6.5%)	
	Origin	1 (1.8%)	0 (0.0%)	1 (3.2%)	
WSS_min_	Downstream	21 (38.2%)	9 (37.5%)	12 (38.7%)	0.927
	Terminal	20 (36.4%)	10 (41.7%)	10 (32.3%)	
	Origin	11 (20.0%)	5 (20.8%)	6 (19.4%)	
	Upstream	3 (5.4%)	0 (0.0%)	3 (9.6%)	
Pressure_max_	Origin	37 (67.3%)	17 (70.8%)	20 (64.5%)	0.620
	Origin-to-M_up_	15 (27.3%)	7 (29.2%)	8 (25.8%)	
	Apex	1 (1.8%)	0 (0.0%)	1 (3.2%)	
	Downstream	1 (1.8%)	0 (0.0%)	1 (3.2%)	
	Terminal	1 (1.8%)	0 (0.0%)	1 (3.2%)	
Pressure_min_	Downstream	39 (70.9%)	16 (66.7%)	23 (74.2%)	0.542
	Terminal	14 (25.5%)	8 (33.3%)	6 (19.4%)	
	Origin	2 (3.6%)	0 (0.0%)	2 (6.4%)	

*WSS, wall shear stress; WSS_max_, the maximum value of WSS; WSS_min_, the minimum value of WSS; Pressure_max_, the maximum value of pressure; Pressure_min_, the minimum value of pressure; M_up_, the middle point of the upstream section; M_down_, the middle point of the downstream section*.

In addition, to verify whether the distribution of specific indices may vary between different plaque locations, we further stratified the plaques according to their locations of the upper side, lower side, and both sides. However, we observed no significant difference between the three subgroups of plaque locations (Table S6).

### Correlation Between Specific Indices and Percent Stenosis

**Figure 2:**
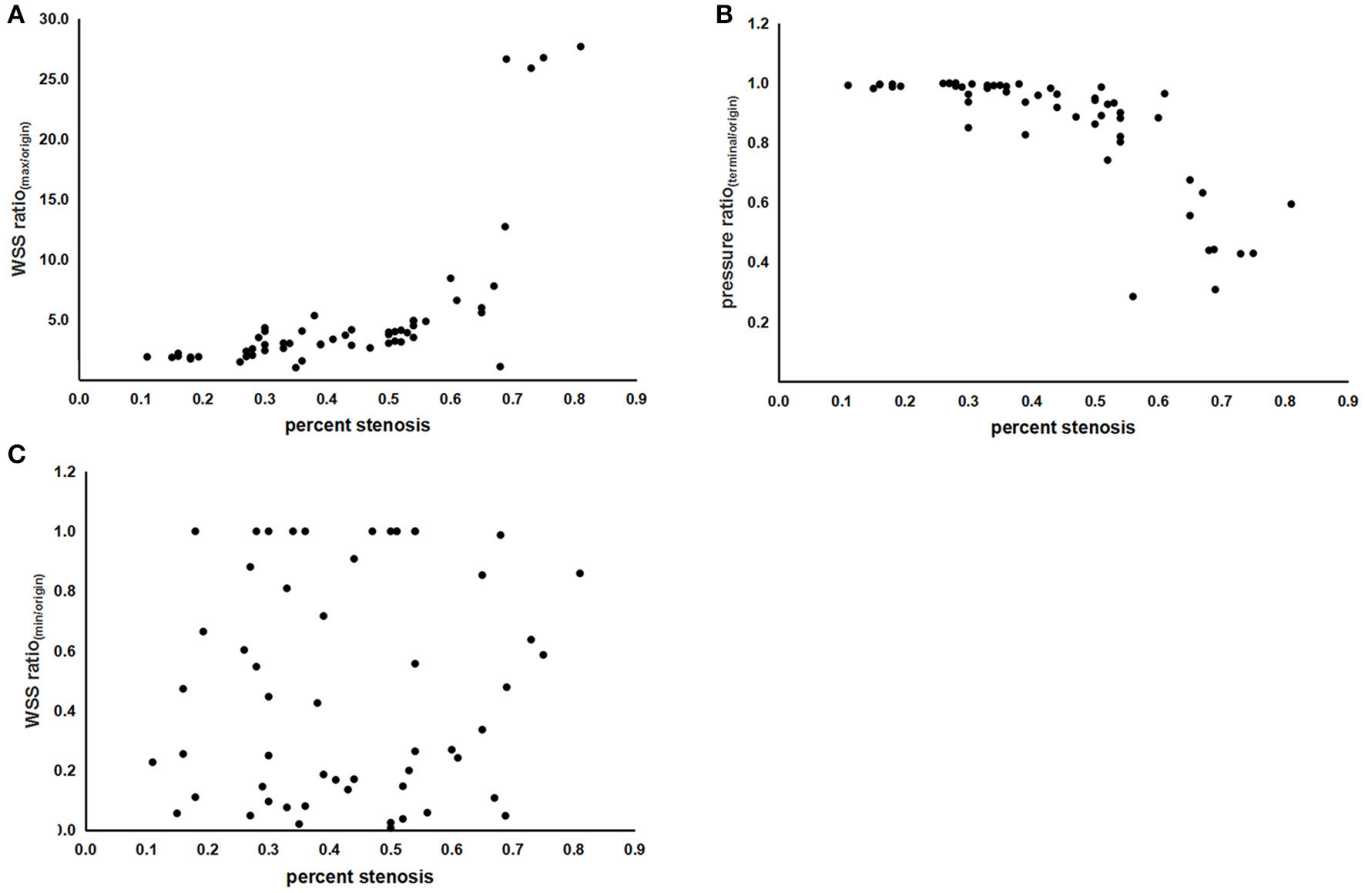
The scatterplots of correlations between indices of hemodynamic forces and percent stenosis. **(A)** Correlation between the WSS ratio_(max/origin)_ and percent stenosis. **(B)** Correlation between the pressure ratio_(terminal/origin)_ and percent stenosis. **(C)** Correlation between the WSS ratio_(min/origin)_ and percent stenosis. WSS, wall shear stress; WSS_max_, the maximum value of WSS; WSS_min_, the minimum value of WSS; WSS_origin_, the value of WSS at the origin point; pressure_terminal_, the value of pressure at the terminal point; pressure_origin_, the value of pressure at the origin point.

As shown in Table 2 and Figures 2A,B, both the WSS ratio_(max/origin)_ (positively) and the pressure ratio_(terminal/origin)_ (negatively) changed quadratically with respect to the percent stenosis with an inflection point around 50%. Subsequently, we divided the data into two groups according to the 50% stenosis and performed Spearman's rank correlation for the two groups, respectively. Adjusted by age and sex, the correlations were significant for the two indices. Furthermore, both of the indices showed different coefficients for the two stenosis severity, with larger *r*_s_ of stenosis ≥50% subgroups, compared with the stenosis <50% subgroups. For WSS ratio_(min/origin)_, we did not find the correlation to be significant (Figure 2C).

**Table 2:** Results of Spearman's rank correlation between indices of hemodynamic forces and percent stenosis.

**Indices of hemodynamic forces**	**Percent stenosis**	**Median (range interquartile)**	***p-*Value**	***r*_**s**_**
WSS ratio_(max/origin)_	<50%	2.61 (1.91, 3.20)	< 0.001	0.598
	≥50%	4.86 (3.86, 7.94)	< 0.001	0.779
Pressure ratio_(terminal/origin)_	<50%	0.99 (0.96, 0.995)	0.003	−0.522
	≥50%	0.81 (0.53, 0.91)	< 0.001	−0.747
WSS ratio_(min/origin)_	—	0.47 (0.66, 0.87)	0.651	0.063

*WSS, wall shear stress; WSS_max_, the maximum value of WSS; WSS_min_, the minimum value of WSS; WSS_origin_, the value of WSS at the origin point; pressure_terminal_, the value of pressure at the terminal point; pressure_origin_, the value of pressure at the origin point*.

**Figure 3:**
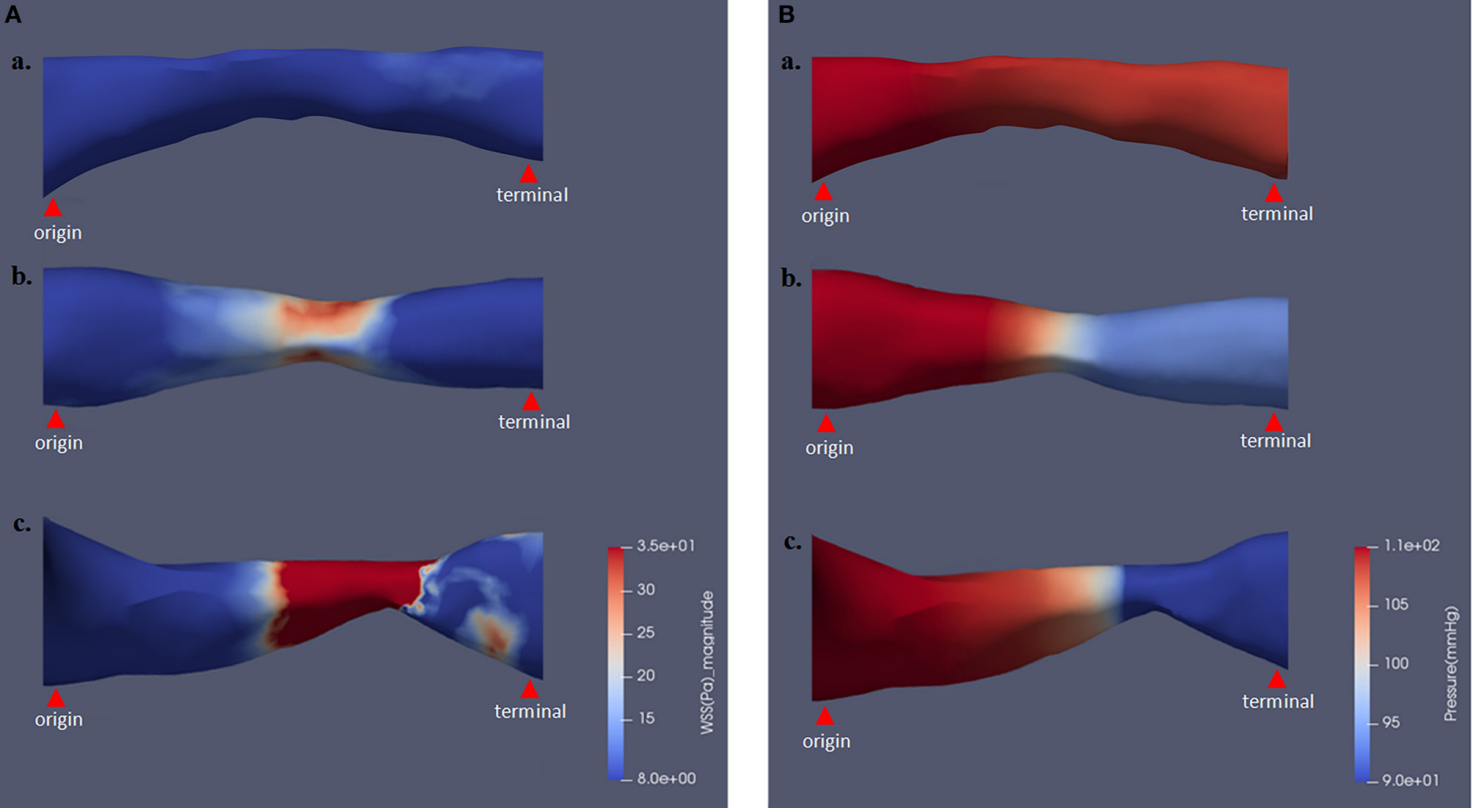
The magnitude and distribution of WSS and pressure varying with different stenosis severity. Panels **(A)** and **(B)**, respectively, show the results of WSS and pressure contour maps. Percent stenosis values of subfigures subfigures (a) and (b) were 47.1, 56.3, and 70.0%, respectively. WSS, wall shear stress.

As shown in Figures 3A,B, the magnitude and distribution of WSS and pressure varied with aggravation of ICAS, percent stenosis of which was 47.1, 56.3, and 70.0% for subfigures a–c, respectively.

### Correlation Between WSS_**max**_, Pressure Ratio_**(terminal/origin)**_, and Pressure Drop_**(origin-to-terminal)**_

**Figure 4:**
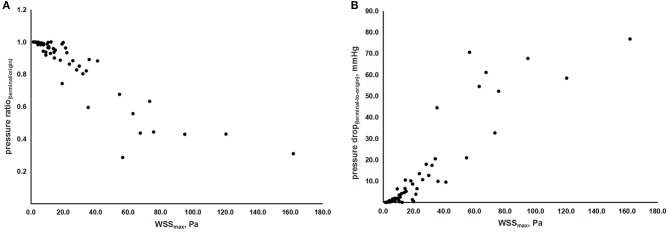
The scatterplots of correlations between indices of hemodynamic forces. **(A)** Correlation between WSS_max_ and pressure ratio_(terminal/origin)_. **(B)** Correlation between WSS_max_ and pressure drop_(origin-*to-terminal*)_. WSS, wall shear stress; WSS_max_, the maximum value of WSS; pressure_terminal_, the value of pressure at the terminal point; pressure_origin_, the value of pressure at the origin point.

Furthermore, by this TOF-MRA-based CFD model, we also evaluated the correlation between indices of hemodynamic forces. Figures 4A,B, show the correlation between WSS_max_, pressure drop_(terminal-*to-origin*)_, and pressure ratio_(terminal/origin)_, with adjustment of age and sex. Therein, we observed a linear relationship between WSS_max_ and the absolute and relative value of pressure drop, as indicated by the coefficients [*r*_s_ = 0.893 (*p* < 0.001) and *r*_s_ = −0.879 (*p* < 0.001), respectively].

## Discussion

In this study, we proposed the potential value of routine TOF-MRA-based cerebral blood flow simulation in hemodynamic assessment of ICAS. For WSS and pressure, we observed the certain trends of longitudinal distribution and changes around atherosclerotic lesions, as well as the correlation between WSS and pressure drop. Notably, the progression of stenosis severity had a significant impact on the magnitude of WSS and pressure, especially when percent stenosis ≥50%.

Previous studies on carotid artery revealed that plaques often ruptured at certain regions under high WSS (16–19), suggesting that the rupture of plaques may be attributed to the high shear rate of blood flow at the region of most severe stenosis. Our study also showed that WSS_max_ mostly occurred within the upper half of upstream sections including the apex points, probably indicating the certain region with high risk of plaque rupture in ICAS. One previous study reported that low WSS could induce the differentiation of an atherogenic endothelial phenotype and the formation of early atherosclerotic lesion through the regulation of endothelial gene expression (20). From our investigation, we observed that WSS_min_ correlated with the downstream section of atherosclerotic lesion in space. Due to the previous study revealing that coronary plaque deposition frequently occurs near low WSS regions (21), this observation indicates the possibility that WSS_min_ may also involve in the ICAS progression, in the direction from the proximal to distal side.

Besides WSS in tangential direction, as the hemodynamic force acting perpendicularly to the lateral wall, pressure also plays an important role in plaque destabilization (5). It has been reported that upstream-ruptured plaques in the carotid artery were associated with a higher pressure drop between the upstream and the downstream shoulder sections (22). The current study showed that the upper half section (between M_up_ and M_down_) sustained the majority of total pressure drop, implying that this region carries a significantly larger mechanical load and may be at a higher risk of plaque rupture in ICAS.

Predicting the critical indices of hemodynamic forces such as WSS_max_ and pressure drop, according to the progression of stenotic severity, can be beneficial for assessing the functional severity of ICAS. We found that the indices of WSS ratio_(max/origin)_ and pressure ratio_(terminal/origin)_ showed a significant correlation with percent stenosis, in a non-linearly proportional manner. Moreover, the scatterplots and the *r*_s_ for each subgroup indicated a threshold effect for the two indexes, suggesting that the relative value of WSS_max_ and pressure drop may increase with percent stenosis in a more dramatic way at ≥50% diameter stenosis. This threshold effect was in accordance with the finding that symptomatic ICAS patients with percent stenosis ≥50% had higher risk of stroke recurrence compared to those with percent stenosis <50% (4). Our results explain the finding from a mechanical viewpoint that percent stenosis significantly influences the hemodynamic forces in ICAS patients, leading to consequent increasing risk of plaque rupture, especially when percent stenosis is ≥50%, indicating the necessity to conduct hemodynamic assessment among this population.

It is worth noting that the index of pressure ratio_(terminal/origin)_ in our study was similar to the recently proposed index of fractional pressure ratio by Liu et al., who have demonstrated that for symptomatic ICAS, it was a good approximation of fractional flow reserve, the gold standard for assessing the physiological significance of coronary stenotic lesions (8, 23). Moreover, one recent study has demonstrated the close correlation between translesional pressure ratio, translesional WSS ratio, and stroke relapse in symptomatic ICAS patients (6). Our study initially demonstrates that the two indexes were not altered significantly until stenosis severity reached 50%. Furthermore, the scatterplots also show the inter-subject variability, indicating the fact that the hemodynamic damage of ICAS was partly determined by percent stenosis, as proved by previous studies on coronary heart disease (24, 25). Other factors may also involve, such as the presence of collaterals and the plaque characteristics (26).

From the proposed TOF-MRA-based CFD model, we also found that the WSS_max_ was linearly correlated with pressure ratio_(terminal/origin)_ and pressure drop_(terminal−to−origin)_ in ICAS, accordingly with the result in coronary artery (27). Since WSSmax values beyond a critical value can induce atherosclerotic inflammation (6), our results also indicated that the plaque vulnerability induced by WSS_max_ may be accompanied with the aggravation of mechanical load, and there was a possibility that plaque vulnerability and hypoperfusion collaboratively participate in the risk of stroke in ICAS.

In the current study, we demonstrated the utility of routine performed TOF-MRA in hemodynamic assessment of ICAS. The decreased sensitivity of TOF-MRA to disturbed and slow flow has been shown, which may limit its application in severe stenosis (28). Instead of the hypoperfusion in severe stenosis, plaque rupture caused by mechanical destruction acts as the major pathological process of stroke in mild to moderate stenosis of ICAS, and it is therefore of clinical value to detect plaque vulnerability, in other words, the imbalance between plaque strength and hemodynamic forces imposing on plaques (29). However, in spite of the high-resolution MRI for evaluation of plaque composition, there is still a lack of methods to elucidate the other aspect. From this perspective, this proposed TOF-MRA-based CFD model can be appropriate for risk evaluation, especially for mild to moderate stenosis.

This study has certain limitations. First, the measurements performed in a plane inherently ignore the plaque morphology out of the chosen plane, which may affect the obtained indices. Second, there is still a lack of established method to calculate the mean blood flow volume of precerebral arteries as the inlet condition. The computation method we used to calculate mean velocity referred to the calculation of mean arterial pressure to achieve an approximate value of time-averaged mean flow velocity was in accordance with previous studies (30, 31). Third, to only evaluate one of the aspects involving in the process of plaque rupture such as the external mechanical forces of WSS and pressure cannot fully address the risk of stroke, especially in the absence of plaque properties reflected by high-resolution MRI, which also prevented us from identifying the atheromatous lesions in a more rigorous way (32). Therefore, to further perform the risk prediction, the evaluation of hemodynamics based on high-resolution MRI is desired from further studies. Fourth, the sample size of this study is relatively small. However, it has clarified the characteristics of hemodynamic forces of ICAS and the non-linear relationship between percent stenosis and the specific indices, which can be verified with larger sample sizes in the future.

## Conclusions

In this study, we performed patient-specific description of the magnitude and distribution of WSS and pressure in realistic geometries of ICAS, which has the potential value to facilitate more accurate risk stratification and more effective prevention for ICAS patients. Meanwhile, we demonstrated the feasibility of a novel radiation- and contrast-free method using routine TOF-MRA-based CFD models, to non-invasively quantify hemodynamic indices of ICAS. Further studies need to verify the roles of hemodynamic forces in governing the stroke risk in ICAS patients and to test indices of the TOF-MRA-based CFD model on accuracy.

## Data Availability Statement

The datasets generated for this study are available on request to the corresponding author.

## Ethics Statement

This study was approved by the ethics committee of Beijing Tiantan Hospital according to the principles expressed in the Declaration of Helsinki. The relevant Judgement's reference number is: KYSB2016-147.

## Author Contributions

YoW, YiW, and LL designed the research protocol and proposed the method of measurement for hemodynamic forces. JL and HQ proposed and designed the CFD model for this study. ZChen wrote the manuscript. HQ and JJ collected the MRA images. BW, ZCheng, XL, and ZChen reconstructed the 3D artery from the MRA images and transferred the 3D artery into meshes. LJ and HQ performed the measurement of hemodynamic forces. YJ and ZChen performed the statistical analysis.

### Conflict of Interest

The authors declare that the research was conducted in the absence of any commercial or financial relationships that could be construed as a potential conflict of interest.

## Abbreviations

M_up_: the middle point of the upstream section
M_down_: the middle point of the downstream section.
