# Supplementary material for: Characteristics of Wall Shear Stress and Pressure of Intracranial Atherosclerosis Analyzed by a Computational Fluid Dynamics Model: A Pilot Study
Source: Front Neurol. 2020 Jan 17;10:1372. doi: 10.3389/fneur.2019.01372 (PMC6978719; doi:10.3389/fneur.2019.01372)
Supplement: Supplementary file 1 [file Table_1.DOC]

**Supplemental table 1.** Baseline characteristics of the patients and the lesions

| Patients characteristics (n=22) | | |
| --- | --- | --- |
| Age, mean (±SD), y | | 65.5 (± 11.5) |
| Sex, male, n (%) | | 12 (54.5%) |
| Lesion characteristics (n=55) | | |
| Percent stenosis, mean (±SD) |  | 43.2% (± 17.3%) |
| Number of stenosis in M1 segments, n (%) |  | 26 (47.3%) |
| Number of percent stenosis ≥ 50%, n (%) | | 25 (43.6%) |

**Supplemental table 2. Magnitude of WSS and pressure at defined points and sections**

| Defined points or sections | External mechanical parameters, Median ([range interquartile](http://www.baidu.com/link?url=BKV1OB6Zu-vU3mhLZBqheSrCeW-k0s-3MCo4jsGih5AUcWhQE2ZDqrUcVrcVaPnavw_PiS-eUmv8IuBq_wxs_TGQnilJ1rscIDtkHDkLCU5PXVhXUzerRVl6DC02Q8Ne)) | | |  |
| --- | --- | --- | --- | --- |
|  | WSS, pa | Pressure, mmHg | Pressure drop, mmHg | Number of  positive / negative values |
| Origin | 3.29 (2.03, 5.23) | 98.14 (83.55, 106.80) | **--** | **--** |
| M_up_ | 5.61 (3.03, 9.01) | 97.06 (82.26, 105.61) | **--** | **--** |
| Apex | 9.27 (5.70, 18.15) | 84.28 (64.03, 99.04) | **--** | **--** |
| M_down_ | 3.08 (1.85, 4.17) | 80.75 (50.71, 97.42) | **--** | **--** |
| Terminal | 2.18 (0.57, 5.23) | 80.77 (50.78, 97.36) | **--** | **--** |
| Maximum value | 13.94 (7.18, 31.04) | 98.14 (83.57, 106.80) | **--** | **--** |
| Minimum value | 1.14 (0.58, 2.58) | 80.75 (50.69, 97.23) | **--** | **--** |
| Origin-M_up_ | **--** | **--** | 0.30 (0.05, 1.25) | 45/10 |
| M_up_-apex | **--** | **--** | 1.62 (0.39, 8.69) | 51/4 |
| Apex-M_down_ | **--** | **--** | 1.21 (0.24, 3.44) | 54/1 |
| M_down_-terminal | **--** | **--** | -0.02 (-0.07, 0.01) | 18/37 |

Abbreviations: WSS, wall shear stress; M_up_, the middle point of the upstream section; M_down_, the middle point of the downstream section.

**Supplemental table 3.** Results of repeated measurements for magnitude of WSS at each point.

|  | *p* value | Adjusted *p* value* |
| --- | --- | --- |
| WSS_apex_ vs WSS_orgin_ | ﹤0.001 | ﹤0.001 |
| WSS_apex_ vs WSS_Mup_ | ﹤0.001 | ﹤0.001 |
| WSS_apex_ vs WSS_Mdown_ | ﹤0.001 | ﹤0.001 |
| WSS_apex_ vs WSS_terminal_ | ﹤0.001 | ﹤0.001 |
| WSS_Mup_ vs WSS_orgin_ | ﹤0.001 | ﹤0.001 |
| WSS_Mup_ vs WSS_Mdown_ | 0.002 | 0.021 |
| WSS_Mup_ vs WSS_terminal_ | ﹤0.001 | ﹤0.001 |
| WSS_orgin_ vs WSS_Mdown_ | 0.175 | 1.000 |
| WSS_orgin_ vs WSS_Terminal_ | 0.103 | 1.000 |
| WSS_Mdown_ vs WSS_terminal_ | 0.003 | 0.028 |

Abbreviations: WSS, wall shear stress; M_up_, the middle point of the upstream section; M_down_, the middle point of the downstream section; WSS_origin_, the value of WSS at the origin point; WSS_Mup_, the value of WSS at the M_up_ point; WSS_apex_, the value of WSS at the apex point; WSS_Mdown_, the value of WSS at the M_down_ point; WSS_terminal_, the value of WSS at the terminal point.

* *p* value was adjusted by Bonferroni correction

**Supplemental table 4.** Results of repeated measurements for magnitude of pressure at each point.

|  | *p* value | Adjusted *p* value* |
| --- | --- | --- |
| Pressure_apex_ vs Pressure_orgin_ | ﹤0.001 | ﹤0.001 |
| Pressure_apex_ vs Pressure_Mup_ | 0.002 | 0.017 |
| Pressure_apex_ vs Pressure_Mdown_ | ﹤0.001 | ﹤0.001 |
| Pressure_apex_ vs Pressure_terminal_ | ﹤0.001 | 0.001 |
| Pressure_Mup_ vs Pressure_orgin_ | 0.020 | 0.203 |
| Pressure_Mup_ vs Pressure_Mdown_ | ﹤0.001 | 0.001 |
| Pressure_Mup_ vs Pressure_terminal_ | ﹤0.001 | 0.001 |
| Pressure_orgin_ vs Pressure_Mdown_ | ﹤0.001 | 0.001 |
| Pressure_orgin_ vs Pressure_terminal_ | ﹤0.001 | 0.001 |
| Pressure_Mdown_ vs Pressure_terminal_ | 0.148 | 1.000 |

Abbreviations: M_up_, the middle point of the upstream section; M_down_, the middle point of the downstream section; pressure_origin_, the value of pressure at the origin point; pressure_Mup_, the value of pressure at the M_up_ point; pressure_apex_, the value of pressure at the apex point; pressure_Mdown_, the value of pressure at the M_down_ point; pressure_terminal_, the value of pressure at the terminal point.

* *p* value was adjusted by Bonferroni correction

**Supplemental table 5.** Results of repeated measurements for magnitude of WSS at each location.

|  | *p* value | Adjusted *p* value* |
| --- | --- | --- |
| Pressure drop_(Mup-to-apex)_ vs Pressure drop_(origin-to-Mup)_ | ﹤0.001 | ﹤0.001 |
| Pressure drop_(Mup-to-apex)_ vs Pressure drop_(Mdown-to-terminal)_ | ﹤0.001 | ﹤0.001 |
| Pressure drop_(apex-to-Mdown)_ vs Pressure drop_(origin-to-Mup)_ | ﹤0.001 | ﹤0.001 |
| Pressure drop_(apex-to-Mdown)_ vs Pressure drop_(Mdown-to-terminal)_ | ﹤0.001 | ﹤0.001 |
| Pressure drop_(Mup-to-apex)_ vs Pressure drop_(apex-to-Mdown)_ | 0.029 | ﹤0.001 |
| Pressure drop_(origin-to-Mup)_ vs Pressure drop_(Mdown-to-terminal)_ | 0.003 | 0.017 |

Abbreviations: M_up_, the middle point of the upstream section; M_down_, the middle point of the downstream section.

* *p* value was adjusted by Bonferroni correction

**Supplemental table 6.** Distribution of specific indices at defined points and sections stratified by different plaque locations (p values for the results of chi-square test indicate whether the most common location of the total distribution become significantly different after grouping)

| Specific indices of hemodynamic forces | Defined points and sections | In total | Grouped by plaque locations | | | *p* value |
| --- | --- | --- | --- | --- | --- | --- |
|  |  |  | Upper-side | Lower-side | Both sides |  |
| WSS_max_ | apex | 29 (52.7%) | 7 (38.9%) | 18 (64.3%) | 4 (44.4%) | 0.12 |
|  | M_up_-to-apex | 22 (40.0%) | 10 (55.6%) | 7 (25.0%) | 5 (55.6%) |  |
|  | M_down_ | 3 (5.5%) | 0 (0.0%) | 3 (10.7%) | 0 (0.0%) |  |
|  | origin | 1 (1.8%) | 1 (5.6%) | 0 (0.0%) | 0 (0.0%) |  |
| WSS_min_ | downstream | 21 (38.2%) | 8 (44.4%) | 11 (39.3%) | 2 (22.2%) | 0.37 |
|  | terminal | 20 (36.4%) | 5 (27.8%) | 11 (39.3%) | 4 (44.4%) |  |
|  | origin | 11 (20.0%) | 5 (27.8%) | 5 (17.9%) | 1 (11.1%) |  |
|  | upstream | 3 (5.4%) | 0 (0.0%) | 1 (3.6%) | 2 (22.2%) |  |
| Pressure_max_ | origin | 37 (67.3%) | 11 (61.1%) | 20 (71.4%) | 6 (66.7%) | 0.50 |
|  | origin-to-M_up_ | 15 (27.3%) | 6 (33.3%) | 7 (25.0%) | 2 (22.2%) |  |
|  | apex | 1 (1.8%) | 0 (0.0%) | 1 (3.6%) | 0 (0.0%) |  |
|  | downstream | 1 (1.8%) | 1 (5.6%) | 0 (0.0%) | 0 (0.0%) |  |
|  | terminal | 1 (1.8%) | 0 (0.0%) | 0 (0.0%) | 1 (11.1%) |  |
| Pressure_min_ | downstream | 39 (70.9%) | 11 (61.1%) | 22 (78.6%) | 6 (66.7%) | 0.35 |
|  | terminal | 14 (25.5%) | 6 (33.3%) | 6 (21.4%) | 2 (22.2%) |  |
|  | origin | 2 (3.6%) | 1 (5.6%) | 0 (0.0%) | 1 (11.1%) |  |

Abbreviations: WSS, wall shear stress; WSS_max_, the maximum value of WSS; WSS_min_, the minimum value of WSS; Pressure_max_, the maximum value of pressure; Pressure_min_, the minimum value of pressure; M_up_, the middle point of the upstream section; M_down_, the middle point of the downstream section.
